# Retraction: Exploring determinants of early marriage among women in Bangladesh: A multilevel analysis

**DOI:** 10.1371/journal.pone.0358825

**Published:** 2026-09-22

**Authors:** The PLOS One Editors

Following publication of this article [1], concerns were raised regarding the suitability of the study design to address the stated aims of the study. The *PLOS One* Editors discussed the following concerns with the corresponding author and consulted two members of the *PLOS One* Editorial Board:

## Selection of participant sample

A concern was raised that the early marriage definition used in [1] is not consistent with international standards [2] which define the prevalence of early marriage as the percentage of all (married and unmarried) women aged 20−24 who married before age 18. In this article [1], early marriage prevalence is calculated as the percentage of ever-married women aged 18−49 who married before age 18. Additionally, the ever-married participant sample in [1] further excluded unmarried women, thereby potentially excluding those who experienced early marriage but are now unmarried. It was noted that estimates for percentages of women married by age in the Bangladesh Demographic and Health Survey 2017−18 report do not exclude never-married women [3].

In response to the concerns, the corresponding author stated that for the purposes of analyzing determinants using multilevel modeling, adherence to the Sustainable Development Goals (SDG) age restriction (20–24 years) [2] was not required and using a larger age range allowed for a greater sample size to support the hierarchical modeling methodology chosen. They also stated that all divorced, separated, or widowed women were excluded because information on husband-related variables (education and occupation) was unavailable.

The consulting members of the Editorial Board noted that it is valid to include only married women in an analysis of determinants of age at marriage, but for estimating prevalence, unmarried women should be included in the denominator; they advised that the reporting of “prevalence” in [1] may be misleading, noting that the percentage of ever-married women aged 18–49 who were married before the age of 18 as reported in [1] is not equivalent to a current national prevalence estimate nor comparable to SDG, UNICEF, or DHS early marriage indicator reporting. They further noted that age-group effects should be interpreted with caution, and one Editorial Board member advised that in this study, exclusion of unmarried women from the participant sample, in combination with the multi-generational sample, resulted in an overestimation of early marriage prevalence in younger women and misleading age covariate results in the multilevel model reported in Table 2.

## Selection of covariates

A concern was raised about the inclusion of household-level and community-level factors as determinants of early marriage among women, as these data reflect the husband’s household and not the woman’s family prior to marriage, and household head roles often shift within families over time.

The corresponding author stated these covariates were selected to capture contextual household-level influences, consistent with prior multilevel studies.

The consulted Editorial Board members considered that family- and community-level characteristics are not appropriate variables to be analyzed as causal or pre-marital determinants since these data do not reflect the woman’s natal household and community information prior to marriage, and therefore are limited to interpretation only as potential correlates of early marriage status. It was further advised that the discussion and recommendations around “empowering older household heads” appear over-interpreted.

In light of the above unresolved concerns about the study design, the interpretation of the reported results, and the validity of the conclusions, the *PLOS One* Editors retract this article.

All authors did not agree with the retraction.
